# RETRACTED: Ndaguba et al. Operability of Smart Spaces in Urban Environments: A Systematic Review on Enhancing Functionality and User Experience. *Sensors* 2023, *23*, 6938

**DOI:** 10.3390/s26144439

**Published:** 2026-07-13

**Authors:** Emeka Ndaguba, Jua Cilliers, Sumita Ghosh, Shanaka Herath, Eveline Tancredo Mussi

**Affiliations:** 1 Centre for Development Support, University of the Free State, Bloemfontein 9301, South Africa; 2 School of Built Environment, Faculty of Design Architecture and Building, University of Technology Sydney, Ultimo, NSW 2007, Australia; jua.cilliers@uts.edu.au (J.C.); sumita.ghosh@uts.edu.au (S.G.); shanaka.herath@uts.edu.au (S.H.); evelinetancredo.mussi@uts.edu.au (E.T.M.); 3 Unit for Environmental Sciences and Management, North-West University, Potchefstroom 2531, South Africa

The journal retracts the article titled, “Operability of Smart Spaces in Urban Environments: A Systematic Review on Enhancing Functionality and User Experience” [1], cited above.

Following publication, concerns were brought to the attention of the Editorial Office regarding the accuracy of the cited sources and scientific terms presented in this publication [1].

In accordance with the journal’s standard procedures, the Editorial Office and Editorial Board conducted an investigation that confirmed the presence of a significant number of non-existent references as well as misrepresentation of the references and scientific terms. As a consequence, the Editorial Bord lost confidence in the reliability of the overall findings and has decided to retract this publication in accordance with MDPI’s retraction policy. 

This retraction was approved by the Editor-in-Chief of the journal *Sensors*.

The authors have been informed of this retraction and have indicated their disagreement with this decision.
